# Long-term safety and efficacy of deferasirox (Exjade®) for up to 5 years in transfusional iron-overloaded patients with sickle cell disease

**DOI:** 10.1111/j.1365-2141.2011.08720.x

**Published:** 2011-05-19

**Authors:** Elliott Vichinsky, Françoise Bernaudin, Gian Luca Forni, Renee Gardner, Kathryn Hassell, Matthew M Heeney, Baba Inusa, Abdullah Kutlar, Peter Lane, Liesl Mathias, John Porter, Cameron Tebbi, Felicia Wilson, Louis Griffel, Wei Deng, Vanessa Giannone, Thomas Coates

**Affiliations:** 1 Children's Hospital and Research Center at OaklandOakland, CA, USA; 2 Department of Pediatrics, Center Hospitalier Intercommunal CréteilCréteil, France; 3 Centro della MicrocitemiaOspedale Galliera, Genoa, Italy; 4 Louisiana State University Health Sciences CenterNew Orleans, LA; 5 University of Colorado Health Sciences CenterDenver, CO; 6 Children's Hospital BostonBoston, MA, USA; 7 Evelina Children's Hospital, Guy's and St Thomas' Hospital NHS TrustLondon, UK; 8 Medical College of GeorgiaAugusta, GA; 9 Children's Healthcare of Atlanta at EglestonAtlanta, GA; 10 Loma Linda University Medical CenterLoma Linda, CA, USA; 11 University College LondonLondon, UK; 12 St Joseph's Children's Hospital of TampaTampa, FL; 13 University of South AlabamaMobile, AL; 14 Novartis Pharmaceuticals CorporationEast Hanover, NJ; 15 Children's Hospital Los AngelesLos Angeles, CA, USA

**Keywords:** deferasirox, Exjade, oral iron chelator, sickle cell disease, iron overload

## Abstract

To date, there is a lack of long-term safety and efficacy data for iron chelation therapy in transfusion-dependent patients with sickle cell disease (SCD). To evaluate the long-term safety and efficacy of deferasirox (a once-daily oral iron chelator), patients with SCD completing a 1-year, Phase II, randomized, deferoxamine (DFO)-controlled study entered a 4-year extension, continuing to receive deferasirox, or switching from DFO to deferasirox. Average actual deferasirox dose was 19·4 ± 6·3 mg/kg per d. Of 185 patients who received at least one deferasirox dose, 33·5% completed the 5-year study. The most common reasons for discontinuation were withdrawal of consent (23·8%), lost to follow-up (9·2%) and adverse events (AEs) (7·6%). Investigator-assessed drug-related AEs were predominantly gastrointestinal [including nausea (14·6%), diarrhoea (10·8%)], mild-to-moderate and transient in nature. Creatinine clearance remained within the normal range throughout the study. Despite conservative initial dosing, serum ferritin levels in patients with ≥4 years deferasirox exposure significantly decreased by −591 μg/l (95% confidence intervals, −1411, −280 μg/l; *P*=0·027; *n*=67). Long-term deferasirox treatment for up to 5 years had a clinically acceptable safety profile, including maintenance of normal renal function, in patients with SCD. Iron burden was substantially reduced with appropriate dosing in patients treated for at least 4 years.

Regular blood transfusion currently represents a supportive therapy to manage the anaemia and vasculopathy associated with sickle cell disease (SCD). In adults, aggressive transfusion to treat and improve organ dysfunction is increasingly used (National Institutes of Health NHLBI, 2002; Wun & Hassell, 2009) and chronic transfusion therapy significantly reduces the risk of primary and secondary stroke in paediatric patients with SCD (Adams *et al*, 1998; Adams & Brambilla, 2005).

As the life expectancy and subsequent transfusion exposure of patients with SCD have increased, so has the risk of transfusional iron overload, which has been associated with morbidity and mortality in SCD (Ballas, 2001). Patients with SCD show differences in iron metabolism and trafficking compared with other haemoglobinopathies; in particular, the effects of inflammatory cytokines in SCD may lead to increased iron levels in reticuloendothelial, macrophage and renal cells, resulting in different tissues and organs being affected by iron overload in SCD (Walter *et al*, 2009). However, regularly transfused patients with SCD still have increased liver iron concentration (LIC), which has been shown to correlate significantly with the volume and duration of transfusions; increased LIC is also associated with liver fibrosis (Harmatz *et al*, 2000; Olivieri, 2001; Brown *et al*, 2009). The number of lifetime transfusions has also been correlated with other markers of iron overload, such as non-transferrin-bound iron and serum ferritin levels (Inati *et al*, 2011). Furthermore, despite patients who are chronically transfused for longer periods being less likely to be hospitalized over a 12-month period, in those patients who were hospitalized, a positive correlation was observed between serum ferritin and hospitalization frequency (Fung *et al*, 2007).

Consequently, guidelines such as those in the USA and UK currently recommend initiating iron chelation therapy in patients with SCD once LIC increases to ≥7 mg Fe/g dry weight, if serum ferritin steady state levels are >1000 μg/l, or if patients have received cumulative transfusions of 120 cc packed red blood cells/kg (USA) or at least 20 top-up transfusions (UK) (National Institutes of Health NHLBI, 2002; Sickle Cell Society, 2008). However, safety and efficacy data have yet to be reported from large prospective clinical trials of any iron chelator in patients with SCD beyond 1 year of follow-up.

Deferasirox is a once-daily, oral iron chelator that has been shown to efficaciously reduce iron burden with a clinically manageable safety profile in patients with β-thalassaemia and other transfusion-dependent anaemias (Cappellini *et al*, 2006; Cappellini *et al*, 2010; Porter *et al*, 2008). During a 1-year, randomized, open-label, Phase II clinical trial, deferasirox demonstrated comparable efficacy to deferoxamine (DFO) and acceptable tolerability in iron-overloaded adult and paediatric patients with SCD (Vichinsky *et al*, 2007). Patients completing this 1-year core study were eligible to enter a 4-year extension. Cumulative 5-year safety and efficacy data of iron chelation therapy with deferasirox in transfusion-dependent patients with SCD are reported here.

## Methods

### Study design and patient population

Male and female patients with SCD aged ≥2 years and with transfusional iron overload were enrolled into the initial core study according to the inclusion/exclusion criteria described previously (Vichinsky *et al*, 2007). Patients who completed the core study and had serum ferritin levels ≥500 μg/l could elect to enter an extension study, which was initially intended to be 3 years, but was extended to 4 years after a protocol amendment. Patients randomized to receive deferasirox in the core study continued with deferasirox during the extension, whereas those who received DFO during the core switched to deferasirox treatment at the start of the extension. Patients continued to receive blood transfusions according to their regimen prior to study entry (chronic or intermittent simple transfusion, or exchange transfusion). Treatment with any regular medication required to treat concomitant conditions, including hydroxycarbamide (hydroxyurea), was permitted during the study.

### Dosing

For patients randomized to deferasirox in the core study, the dose at the start of the extension study was determined by their serum ferritin trends during the core study. In patients with stable or increasing serum ferritin trends, the initial dose could be increased by 5 or 10 mg/kg per d, whereas the dose was maintained in patients who had declining serum ferritin trends during the core. In patients randomized to DFO in the core study, initial deferasirox dose was determined by the treating investigator according to the following guidelines: an initial dose of 20 mg/kg per d was recommended for patients receiving 2–4 units/month (or 7–14 ml/kg per month) of packed red blood cells, although initial doses of 10 or 30 mg/kg per d could be considered for patients with lower or higher transfusion requirements, respectively. During the extension study, dose adjustments in increments of 5 or 10 mg/kg per d were allowed every 3 months based on trends in serum ferritin levels and safety parameters.

### Assessments

All adverse events (AEs) and serious AEs were monitored and recorded regularly by investigators. Details of AEs and their management are reported in accordance with the information provided by the investigating clinician. Laboratory parameters, including haematology, blood chemistry, urine, liver function and renal function analyses were monitored at least monthly; samples were processed at a central laboratory. Assessment of vital signs (height, weight, sitting pulse and sitting blood pressure), auditory and visual functions were regularly performed. In paediatric patients (aged 2–<16 years), additional assessments included those of stature, growth velocity and pubertal development according to Tanner staging (Marshall & Tanner, 1969, 1970). Individual growth curves of paediatric patients were plotted based on the Centers for Disease Control and Prevention growth charts for the US (Kuczmarski *et al*, 2000). Efficacy was evaluated by monthly measurement of serum ferritin levels.

### Statistical analysis

Safety and efficacy data are reported for all patients who received at least one dose of deferasirox during the core or extension studies, unless otherwise stated. The statistical significance of differences in serum ferritin levels from start of deferasirox treatment to end of study was determined using the two-sided Sign test at the significance level α 0·05. Serum ferritin values are reported as median (range). Changes in serum ferritin are reported as median [95% confidence intervals (CI)]. Serum ferritin values at the start of deferasirox for patients who received deferasirox during the core study were taken as the mean of all available pretreatment values. For patients who received DFO during the core study, the mean of the last three available serum ferritin level assessments prior to starting deferasirox was used. Reported end-of-study values for serum ferritin represent the average of the last three available assessments after the start of deferasirox. Average iron intake was derived from the monthly blood intake using the formula: average iron intake (mg/kg/d) = average blood intake (ml/kg/month) × 1·08/28 days. Average iron intake and average actual dose are reported as mean ± SD. Creatinine clearance was calculated based on the serum creatinine level and the last available height and weight measurement prior to the sample date, using the Schwartz formula (Schwartz *et al*, 1987) for paediatric patients and the Cockcroft–Gault formula (Cockcroft & Gault, 1976) for adult patients.

## Results

### Study population

One hundred and ninety-five patients were initially enrolled at 44 sites in Canada, France, Italy, the UK and the USA; 173 completed the core and 163 entered the extension study. Patients who chose not to enter the extension did not have to explain their reasoning. Overall, 185 patients received at least one dose of deferasirox between 27 May 2003 (first patient enrolled) and 31 July 2009 (last patient, last visit). Patient demographics at the start of deferasirox treatment, corresponding to core baseline or start of the extension in patients initially randomized to deferasirox or DFO, respectively, are summarized in Table I. Ninety of 185 patients (48·6%) treated with deferasirox were aged <16 years.

**Table I:** Patient demographics at the start of deferasirox treatment

Characteristic	All patients (*n* =185)
Mean age (range), years	19·2 (3·0–54·0)
Age group, *n* (%)	
<6 years	5 (2·7)
6–<12 years	42 (22·7)
12–<16 years	43 (23·2)
16–<50 years	91 (49·2)
50–<65 years	4 (2·2)
Male:female, *n*	74:111
Caucasian:black:other, *n*	11:167:7
History of splenectomy, *n* (%)	24 (13·0)
Median serum ferritin (range), μg/l	3329 (405–12,901)
Serum ferritin, *n* (%)	
500–1000 μg/l	3 (1·6)
> 1000–2500 μg/l	61 (33·0)
>2500–4000 μg/l	48 (25·9)
>4000 μg/l	73 (39·5)

The 5-year study was completed by 62 patients (33·5%) overall. In total, 71 patients (38·4%) discontinued after withdrawing consent (specific reasons for this were not reported), administrative problems or were lost to follow-up. These patients accounted for the majority (57·7%) of the 123 who discontinued. Discontinuation as a result of AEs was reported in 14 (7·6%) patients overall (Table II).

**Table II:** Patient disposition after the start of deferasirox treatment

Disposition, *n* (%)	Patients (*n* =185)
Completed	62 (33·5)
Discontinued	123 (66·5)
Adverse events	14 (7·6)
Abnormal laboratory value/test procedure	6 (3·2)
Unsatisfactory therapeutic effect	6 (3·2)
No longer requires study drug	9 (4·9)
Protocol violation	3 (1·6)
Subject withdrew consent	44 (23·8)
Lost to follow-up	17 (9·2)
Administrative problems	10 (5·4)
Death	3 (1·6)
Stopped at end of core	7 (3·8)
Stopped at end of extension 1*	4 (2·2)

* Completed 3-year extension study before extension was prolonged to 4 years by protocol amendment

Of the 61 patients who withdrew consent or were lost to follow-up, nine (14·8%) reported AEs within 2 weeks prior to study discontinuation. The AEs were predominantly mild in severity (Table III) and none were suspected to be related to deferasirox treatment.

**Table III:** AEs after start of deferasirox within 2 weeks prior to discontinuation due to withdrawal of consent or loss to follow-up

AE, *n* (%)	Patients (*n*= 185)	Severity
Any AE	9 (4·9)	–
Influenza	1 (0·5)	Moderate
Nasopharyngitis	1 (0·5)	Mild
Staphylococcal infection	1 (0·5)	Moderate
Pain in extremity	2 (1·1)	Mild/moderate
Back pain	1 (0·5)	Mild
Sickle cell crisis	1 (0·5)	Severe
Ear pain	1 (0·5)	Mild
Pyrexia	1 (0·5)	Mild
Excoriation	1 (0·5)	Mild
Increased γ-glutamyltransferase	1 (0·5)	Mild

### Deferasirox dosing and exposure

During the study, patients received an average actual deferasirox dose of 19·4 ± 6·3 mg/kg per d and the median duration of deferasirox treatment was 36·9 months.

### Safety and tolerability

**Fig 1:**
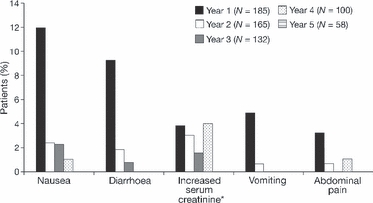
Yearly frequency of most common (≥5% overall) investigator-assessed drug-related AEs after the start of deferasirox. *Reported as an AE by the investigator.

The most common AEs reported in >40% of patients overall were headache (*n* *=*94, 50·8%), sickle cell crisis (*n* =91, 49·2%) and pyrexia (*n* =83, 44·9%). Sickle cell crisis leading to hospitalization was reported in 64 patients (34·6%). Pyrexia was reported on the same day as sickle cell crisis in 15 patients (8·1%). Ninety-three patients (50·3%) reported AEs that investigators suspected to be study drug-related; the most frequently reported (≥5% overall) are listed in Table IV (all investigator-assessed drug-related AEs are listed in Table SI). These were mostly gastrointestinal disorders that were mild-to-moderate, transient in nature and decreased in frequency after the first year (Fig 1). Three patients discontinued the study as a result of gastrointestinal disorders with a suspected relationship to deferasirox treatment [abdominal pain (*n* = 1), acute pancreatitis (*n* = 1) and diarrhoea (*n* = 1)].

**Table IV:** Most common (≥5% overall) investigator-assessed drug-related adverse events after the start of deferasirox

Adverse event, *n* (%)	All patients (*n* =185)
Nausea	27 (14·6)
Diarrhoea	20 (10·8)
Increased serum creatinine*	11 (5·9)
Vomiting	10 (5·4)
Abdominal pain	9 (4·9)

* Increase in serum creatinine assessed by investigators to be clinically significant and reported as an adverse event (AE). No specific parameters were defined for reporting laboratory assessments as AE.

Serious AEs were experienced by 131 patients (70·8%) overall. The most common serious AEs, which were reported in ≥10% of all patients, were sickle cell crisis (*n* =67, 36·2%) and pyrexia (*n* =34, 18·4%). Deferasirox was permanently discontinued in 12 patients (6·5%) following serious AEs. Nine patients (4·9%) had 17 serious AEs that investigators suspected to be related to deferasirox treatment, including gastrointestinal disorders (*n* =3), sickle cell crisis (*n* =2), lymphadenopathy (*n* =1), tinnitus (*n*=1), granuloma (*n* =1), tuberculosis (*n* =1), spontaneous abortion (*n* =1), pulmonary thrombosis (*n* =1), increased alanine aminotransferase (ALT; *n* =1), increased aspartate aminotransferase (AST; *n* =1), increased blood alkaline phosphatase (*n* *=* 1), increased blood amylase (*n* =1), increased blood bilirubin (*n* =1), increased lipase (*n* =1) and increased transaminases (*n* =1). Neither of the patients who had sickle cell crisis with a suspected relationship to deferasirox was receiving the drug at the time of the event. No action with respect to deferasirox dosing was taken following the case of spontaneous abortion; the patient received 20 mg/kg per d deferasirox throughout the extension until discontinuation as a result of an AE (otitis media, not suspected to be related to deferasirox treatment).

AEs led to deferasirox dose adjustment or interruption in 107 patients (57·8%; Table SII); serious AEs led to dose adjustment or interruption in 65 patients (35·1%). Dose adjustments and interruptions were primarily a result of gastrointestinal adverse reactions (*n* =38, 20·5%). Increases in serum creatinine levels (reported by investigators as an AE) led to dose adjustment or interruption in 11 (5·9%) patients overall. Data were available for 37 patients before and after deferasirox dose increases to >30 mg/kg per d during the study; exposure to doses ≤30 mg/kg per d and >30 mg/kg per d was 98·3 and 50·9 patient years, respectively. There were no apparent differences in the incidence or type of AEs before and after the dose increased above 30 mg/kg per d. Similarly, there were no clinically meaningful differences in renal or liver laboratory parameters assessed before and after dose increases above this threshold.

There were three deaths during the study. One patient died as a result of intracranial haemorrhage following a liver transplantation. This patient had underlying liver disease secondary to hepatitis C, in addition to haemosiderosis. After receiving deferasirox for approximately 40 months, the patient was diagnosed with hepatic failure, which was assessed as related to the underlying disease and not to the study drug. Ten days after discontinuing deferasirox as a result of the hepatic failure, as well as associated renal failure, the patient underwent a liver transplantation. The day following the transplantation the patient died as a result of an intracranial haemorrhage. The other two deaths were both due to intraventricular haemorrhage. One patient had a history of transient ischaemic attack and cerebrovascular accident, while the other had Moyamoya disease at the time of enrolment. None of the three deaths was suspected to be related to treatment with deferasirox.

#### Renal parameters

**Fig 2:**
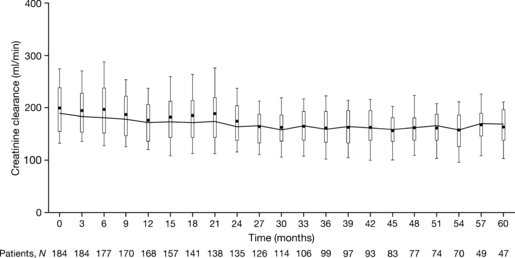
Quarterly creatinine clearance assessments after start of deferasirox treatment. Boxes define the interquartile range. Whiskers extend to the 10 and 90th percentiles. The line connects the median values.

Calculated creatinine clearance remained stable during deferasirox treatment for up to 5 years (Fig 2). Two patients (1·1%) reported reduced creatinine clearance in the >40–≤60 ml/min range. No patients had a reduction of creatinine clearance to <40 ml/min at two consecutive assessments. Ten patients (5·4%) presented with serum creatinine values greater than the upper limit of normal (ULN) and >33% above the value at the start of deferasirox treatment on two consecutive visits during the study: all had serum creatinine levels below the upper limit of normal (ULN) at the start of deferasirox treatment except for one who had received DFO during the core study. This patient had presented with these criteria on multiple occasions while receiving DFO but successfully completed the extension while taking deferasirox; serum creatinine levels were mostly stable during the extension. Two patients discontinued deferasirox because of abnormal renal parameters and one discontinued for reasons that were unclear from the information provided. In three patients, serum creatinine levels normalized following temporary interruptions to deferasirox treatment and these patients went on to complete the study. The remaining three patients completed the study without dose decreases or interruption in response to increases in serum creatinine levels >33% above levels at the start of deferasirox and >ULN; serum creatinine levels returned to normal range in one patient and remained elevated in the other two patients. Acute renal failure was reported in two patients (1·1%) in the deferasirox cohort; both events were assessed to be due to the patients’ underlying condition. In one of these patients, serum creatinine levels were elevated to 123·8 μmol/l and a computed tomography scan revealed a haemorrhagic cyst of the left kidney. Deferasirox was discontinued because of acute renal failure and simultaneous hepatic failure. The patient's subsequent death as a result of intracranial haemorrhage, post-liver transplantation, is described above. In the second patient, serum creatinine levels increased to 97·2 μmol/l during an episode of acute chest syndrome and pneumonia. Deferasirox was continued without alteration and the acute renal failure resolved following concomitant treatment (infusion of normal saline) and the patient completed the study. Three patients (1·6%) had notable abnormalities in total urinary protein:creatinine ratio (>1·0 mg/mg) at two consecutive assessments. In two patients, proteinuria resolved following a temporary dose interruption. The third patient was not receiving deferasirox at the time that the increased urinary protein:creatinine ratio was recorded. There were no reported cases of serious tubular damage (Fanconi syndrome) during deferasirox treatment.

#### Hepatic parameters

Five patients (2·7%), had increased ALT values >5 × ULN on two consecutive assessments during treatment with deferasirox; all had ALT levels >ULN prior to entering the study. A further two patients (1·1%) had ALT values >10 × ULN at two consecutive visits, one at day 582 while receiving 20 mg/kg per d and one at day 932 while receiving 40 mg/kg per d; prior to commencing deferasirox treatment, ALT levels were <ULN in both these patients. ALT levels returned to the normal range in both patients following a temporary interruption in deferasirox treatment and remained at normal levels when the medication was restarted until end of study. Overall, median ALT and AST levels remained constant at normal levels throughout the study. Hepatic failure was reported in one of the five patients with two consecutive ALT values >5 × ULN; when admitted to hospital, ALT and AST levels were elevated to 64 and 124 U/l respectively. Total bilirubin level was 83·8 μmol/l and direct bilirubin was 59·8 μmol/l. The patient had presented with hepatitis C and abnormal liver function tests at the time of enrolment and hepatic failure was not suspected to be related to deferasirox treatment. Deferasirox was discontinued following diagnosis of hepatic failure and the patient underwent liver transplantation. The death of this patient as a result of intracranial haemorrhage post transplantation is described above. Hepatic cirrhosis was reported as a serious AE in one patient and as an AE in a further two patients; the event was not suspected to be related to deferasirox treatment.

#### Renal and hepatic parameters in patients who received concomitant hydroxycarbamide

Twenty patients received concomitant hydroxycarbamide during the study, of whom one experienced increases in serum creatinine >33% above the value at the start of deferasirox and >ULN at two consecutive assessments and one had increases in ALT >5 × ULN at two consecutive visits.

#### Other safety parameters

Neutropenia, defined as absolute neutrophil counts <1·5 × 10^9^/l at two consecutive assessments after the start of deferasirox, was reported in one patient (0·5%). The patient was not receiving deferasirox at the time of the reported neutropenia as serum ferritin levels were <500 μg/l.

Thrombocytopenia, defined as platelet counts <100 × 10^9^/l at two consecutive assessments after the start of deferasirox, was reported in three patients (1·6%). In one of these patients, deferasirox was discontinued prior to death because of intracranial haemorrhage post-liver transplantation (as described above). Thrombocytopenia resolved without alteration of deferasirox treatment in the other two patients, who both completed the study.

Mild lenticular opacities, suspected by investigators to be related to the study drug, were reported in two patients (1·1%). These events were reported during the core phase in both eyes in one patient and during the third year of deferasirox treatment in the right eye of the other patient. Deferasirox dose was 20 and 30 mg/kg per d, respectively, in these patients at time of onset. No action was taken with respect to deferasirox treatment. The condition resolved after 211 days in the first patient, who continued the study until the fifth year, then withdrew consent. The second patient completed the study despite ongoing lenticular opacity.

Nine patients (4·9%) had clinically significant audiometric test abnormalities at the start of deferasirox; three of these patients reported normal results during the study. A further nine patients who had normal audiometric test results at the start of deferasirox developed audiometric test abnormalities during deferasirox treatment. These changes were often transient despite continuation of deferasirox treatment; of the nine patients who developed new, clinically significant audiometric test abnormalities during the study, four continued to present with these abnormalities at the end of study. No action was taken with respect to deferasirox treatment following clinically significant audiometric test abnormalities.

Gastrointestinal haemorrhage was reported in two patients (1·1%) but neither case was suspected to be related to treatment; these events resolved following medical intervention, and treatment with deferasirox was either continued or temporarily interrupted during the event. Rash (of all types) was reported as an AE in 36 patients (19·5%) overall, but was only suspected to be related to deferasirox treatment by investigators in seven patients (3·8%). One patient (0·5%) discontinued deferasirox as a result of rash that investigators assessed to be drug related.

#### Growth and development in paediatric patients

Steady increases in weight and height were noted for 90 paediatric patients aged 2–<16 years. Individual growth curves demonstrated continuous growth of paediatric patients during the study in line with normal trends derived from US clinical growth charts; the majority of paediatric patients demonstrated growth within the 5–95th percentile range. Sexual development, as assessed by Tanner staging for breast development and pubic hair in females and for testes volume and pubic hair in males, progressed as expected for adolescent patients.

### Efficacy

**Fig 3:**
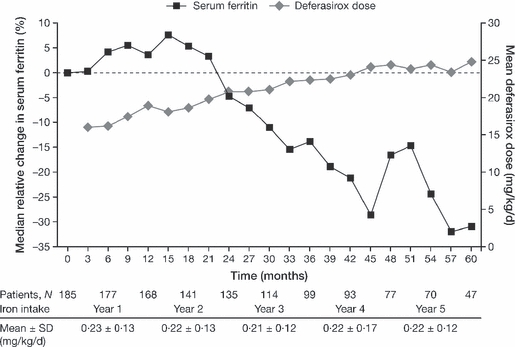
Median relative change in serum ferritin levels and average actual deferasirox dose. Three-monthly values include the last available serum ferritin assessment/dose available for that 3-month period. SD, standard deviation.

Patients continued on their regular transfusion regimen during the study and mean overall iron intake remained relatively stable, with most patients receiving <0·3 mg/kg per d of iron during up to 5 years of deferasirox treatment. For patients who remained in the study, serum ferritin levels steadily decreased with deferasirox treatment for up to 5 years, with the largest decreases observed after the average actual deferasirox dose increased above 20 mg/kg/d (Fig 3). In patients who received deferasirox for at least 4 years, median serum ferritin levels decreased significantly from 3410 μg/l (1082–10,451 μg/l) at the start of deferasirox to 3108 μg/l (88–14,743 μg/l) at end of study; the median absolute change in serum ferritin was −591 μg/l (95% CI, −1411, −280 μg/l; *P*=0·027; *n* *=* 67). For all patients who received at least one deferasirox dose, median serum ferritin levels were 3329 μg/l (405–12,901 μg/l) at start of deferasirox and 3351 μg/l (34–14,743 μg/l) at end of study using last observation carried forward analysis.

**Fig 4:**
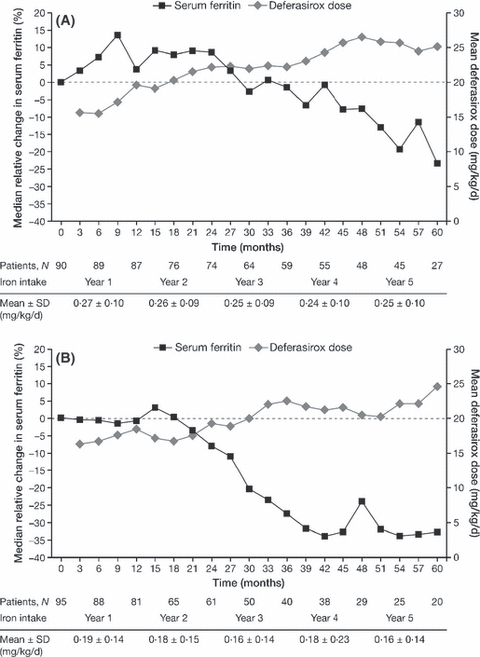
Median relative change in serum ferritin levels from start of deferasirox treatment and average actual deferasirox dose in (A) paediatric and (B) adult patients. Three-monthly values include the last available serum ferritin assessment/dose available for that 3-month period. SD, standard deviation.

The relative decrease in serum ferritin was greater in adult than in paediatric patients. Paediatric patients were initiated on lower deferasirox doses than adult patients (mean average actual dose in months 0–3 was 15·6 ± 6·4 mg/kg per d in paediatric patients and 16·4 ± 6·7 mg/kg per d in adult patients). Paediatric patients, who had a higher annual mean iron intake than adult patients, required increases in average actual deferasirox dose to ≥25 mg/kg per d to achieve a median relative decrease in serum ferritin levels after starting deferasirox, whereas relative decreases in serum ferritin were observed in adult patients at doses of 20 mg/kg per d (Fig 4).

For patients with an available end of study (last observation carried forward) serum ferritin change, 81 of 142 (57·0%) patients in the <0·3 mg/kg per d mean overall iron intake category and 19 of 34 (55·9%) patients in the 0·3–0·5 mg/kg per d mean overall iron intake category had decreases in serum ferritin levels of ≥10% from start of deferasirox treatment to end of study (last day on deferasirox). Two patients had mean overall iron intakes of >0·5 mg/kg per d; both had decreases in serum ferritin levels of ≥10% from start of treatment to end of study.

## Discussion

In patients with SCD, transfusion therapy is frequently initiated in childhood for the prevention of primary and secondary stroke and may be indicated for continued use in adults at high risk of progressive pulmonary, heart, renal and brain injury (Vichinsky, 2001; National Institutes of Health NHLBI, 2002; Wun & Hassell, 2009). The increased life expectancy of patients with SCD has subsequently led to a need for long-term iron chelation therapy to prevent the onset of complications associated with iron overload, but long-term iron chelation data in SCD are currently limited. This is the largest study to date to assess the long-term safety and efficacy of an iron chelator, deferasirox, in patients with SCD.

Of the patients who received at least one dose of deferasirox, 62 (33·5%) completed the extension study. The lack of data from similarly long-term, prospective clinical trials in SCD makes it difficult to assess the significance of the high discontinuation rate with respect to deferasirox treatment. More than half of the total discontinuations were recorded as withdrawal of consent, loss to follow-up or administrative problems, while only 14·1% were due to factors related to therapy (AEs, abnormal laboratory value/test procedure or unsatisfactory therapeutic effect). Only nine of 61 patients who discontinued because of withdrawal of consent or loss to follow-up experienced AEs within 2 weeks prior to discontinuation. The majority of these AEs were mild in severity and none were suspected to be related to deferasirox treatment, suggesting that adverse reactions to the study drug were unlikely to contribute to study discontinuation in these patients. Furthermore, the rates of discontinuation during the 1-year core phase of the study were similar for patients randomized to DFO or deferasirox (Vichinsky *et al*, 2007). A similar profile of reasons for discontinuation, i.e, primarily for reasons other than AEs, has been observed in several shorter-term SCD clinical trials of hydroxycarbamide (Charache *et al*, 1992; Kinney *et al*, 1999; Steinberg *et al*, 2003). Meanwhile, a similarly designed long-term study of deferasirox in patients with β-thalassaemia for up to 5 years reported a completion rate approximately double to that observed for this study (Cappellini *et al*, 2009). Combined, these observations suggest that disease and social factors specific to SCD patient populations may contribute substantially to low study completion rates.

The most frequently reported AEs in this study, such as headache, sickle cell crisis and pyrexia, were either non-specific or considered to be reflective of the underlying SCD rather than of the study drug. As the study design did not include a control arm, it is not possible to assess the impact of the deferasirox treatment on the frequency of sickle cell crises; however, none of the patients interrupted or discontinued deferasirox because of an apparent increase in SCD activity. During the core phase of the study, the frequency of sickle cell crisis was comparable in patients randomized to deferasirox (33·3%) and DFO (31·7%), suggesting that incidence is comparable irrespective of chelator type (Vichinsky *et al*, 2007). High frequencies of sickle cell pain events, particularly in an outpatient context, have been demonstrated by diary studies (Smith *et al*, 2008), whereas data from the Stroke Prevention Trial (STOP) have demonstrated that transfusions only lead to significant reduction in pain events when aggressive, chronic regimens are strictly complied with (Miller *et al*, 2001).

AEs suspected to be related to deferasirox treatment were predominantly gastrointestinal, transient and mild-to-moderate in nature, as observed in the core study and trials of deferasirox in other transfusion-dependent anaemias (Cappellini *et al*, 2006; Porter *et al*, 2008; Cappellini *et al*, 2010; Vichinsky *et al*, 2007). The incidence of rash with a suspected relationship to deferasirox was lower in this study than in other deferasirox trials, although the reason for this is unclear. The frequency of the most common drug-related AEs generally decreased year-on-year, suggesting that tolerability to deferasirox improves with long-term treatment. Although the number of patients is limited (*n* *=* 37), it is encouraging to note that, following dose increases to ≥30 mg/kg per d, there was no apparent change in the safety profile, in line with previous studies (Taher *et al*, 2009).

The prescribing information for deferasirox notes a risk of renal and hepatic impairment, including failure, and gastrointestinal haemorrhage (predominantly in patients with myelodysplastic syndromes). Renal failure, hepatic failure and gastrointestinal haemorrhage were reported in a very small number of cases during the present study and in each case investigators believed the event to be related to patients’ underlying co-morbidities rather than deferasirox treatment. The kidney, as a site of deferasirox toxicity, is of particular relevance to patients with SCD who frequently present with abnormal renal function. Creatinine clearance was relatively stable throughout the study in both cohorts and most of the notable increases in serum creatinine were managed without the need to permanently discontinue deferasirox treatment. Disease-related factors may reduce the sensitivity of serum creatinine and creatinine clearance assessments in evaluating renal function in patients with SCD; however, urinary protein:creatinine ratio was also assessed during the study and notable abnormalities were only reported in three patients. Few patients presented with abnormal ALT levels and significant elevations were transient and manageable. The onset of AEs and clinically significant changes in laboratory parameters did not appear to be related to deferasirox dose or length of exposure.

Almost half of the patients included in this analysis were paediatric patients aged <16 years. Paediatric patients with SCD often have retarded growth and delayed sexual maturation (Platt *et al*, 1984), which may be normalized by long-term transfusion therapy (Wang *et al*, 2005). During long-term deferasirox treatment, most paediatric patients had normal growth compared to a standard US population and sexual development also progressed without evidence of significant delay. As such, the benefits of transfusion on paediatric growth and development in patients with SCD do not seem to be impeded by long-term treatment with deferasirox.

In all patients who completed at least 4 years of deferasirox treatment, the median serum ferritin level was significantly reduced. However, the conservative dosing regimen adopted at the start of deferasirox treatment meant that overall median serum ferritin levels did not decrease below baseline during the first 2 years, when the average actual dose was <20 mg/kg per d. This trend was prolonged in paediatric patients, in whom median serum ferritin levels remained at or above the levels reported at the start of deferasirox for 3 years. This could be reflective of the low initial dosing of deferasirox in paediatric patients during the core study, despite generally higher mean overall iron intake compared with adults, thus suggesting a similar relationship between iron intake and response to a given deferasirox dose to that reported in a β-thalassaemia study (Cohen *et al*, 2008). The pharmacokinetic profile of deferasirox is such that the steady-state exposure is lower in paediatric patients than in adults, meaning paediatric patients with SCD may require higher doses to achieve effective iron chelation (Galanello *et al*, 2006). Efficacy results should also be interpreted with the consideration that start of deferasirox represents different time points in the study depending on whether patients were randomized to receive deferasirox or DFO during the core phase. Fifty-three of the 185 patients included received DFO under controlled trial conditions during the first year of the study; these patients received deferasirox for a maximum of 4 years, rather than 5 years, and had generally lower serum ferritin levels at the start of deferasirox treatment compared with those randomized to receive deferasirox from the onset of the study.

Assessment of the efficacy of deferasirox in reducing iron burden was limited to serum ferritin analysis in this study. Serum ferritin levels can be influenced by inflammatory processes (Piperno, 1998), which are often exacerbated in patients with SCD because of vaso-occlusive crisis and haemolysis, as well as infection (Brittenham *et al*, 1993). Inflammatory responses indicate that patients with SCD have higher levels of cytokines, which affect the synthesis of ferritin and its release into the circulation (Walter *et al*, 2006). Serum ferritin levels in patients with SCD have shown varying degrees of correlation with LIC, which is considered a more direct measure of iron overload, depending on the methods of LIC assessment (Brittenham *et al*, 1993; Karam *et al*, 2008). Although LIC was not assessed during the extension, the maintenance of overall ALT and AST at normal levels throughout the study suggests that liver function did not decrease during deferasirox treatment. Such considerations are important when evaluating iron overload, deferasirox efficacy and dosing strategy specifically in patients with SCD. Iron loading patterns differ in patients with SCD compared with thalassaemia major, not only as a result of disease-associated factors, including chronic inflammation and intravascular haemolysis (Porter, 2009), but also because of different transfusion regimens and frequencies in patients with SCD, which give them a unique iron intake profile.

In conclusion, long-term iron chelation therapy with deferasirox was associated with a manageable safety profile and did not significantly affect kidney function in most adult and paediatric patients with SCD during this study. In patients with SCD who remain on the treatment regimen, deferasirox with appropriate dosing represents an effective long-term treatment to accompany ongoing transfusion therapy, facilitating the prevention of stroke and organ failure by potentially reducing the risk of developing complications of iron overload.

## Supporting information

**Table SI.** Investigator-assessed drug-related adverse events.

**Table SII.** Adverse events leading to deferasirox dose adjustment or interruption, irrespective of study drug relationship.
