# Supplementary material for: Long-term safety and efficacy of deferasirox (Exjade®) for up to 5 years in transfusional iron-overloaded patients with sickle cell disease
Source: Br J Haematol. 2011 May 19;154(3):387–97. doi: 10.1111/j.1365-2141.2011.08720.x (PMC3170481; doi:10.1111/j.1365-2141.2011.08720.x)
Supplement: Supplementary file 1 [file bjh0154-0387-SD1.doc]

***British Journal of Haematology* manuscript BJH-2010-01685**

**Online supplementary information**

**Vichinsky *et al.* Long-term safety and efficacy of deferasirox (Exjade®) for up to 5 years in transfusional iron-overloaded patients with sickle cell disease**

**Table S1. Investigator-assessed drug-related adverse events**

| **Adverse event, *N* (%)** | **All patients (*N* = 185)** |
| --- | --- |
| Any adverse event | 93 (50.3) |
| Nausea | 27 (14.6) |
| Diarrhoea | 20 (10.8) |
| Vomiting | 10 (5.4) |
| Abdominal pain | 9 (4.9) |
| Upper abdominal pain | 7 (3.8) |
| Abdominal discomfort | 6 (3.2) |
| Constipation | 3 (1.6) |
| Abdominal distension | 1 (0.5) |
| Lower abdominal pain | 1 (0.5) |
| Dyspepsia | 1 (0.5) |
| Flatulence | 1 (0.5) |
| Acute pancreatitis | 1 (0.5) |
| Oral paraesthesia | 1 (0.5) |
| Increased serum creatinine* | 11 (5.9) |
| Increased ALT* | 8 (4.3) |
| Increased AST* | 8 (4.3) |
| Increased transaminases* | 4 (2.2) |
| Increased blood urea* | 2 (1.1) |
| Increased urine protein/creatinine ratio* | 2 (1.1) |
| Blood alkaline phosphatase* | 1 (0.5) |
| Increased blood amylase* | 1 (0.5) |
| Increased blood bilirubin* | 1 (0.5) |
| Increased lipase* | 1 (0.5) |
| Abnormal liver function test* | 1 (0.5) |
| Protein urine* | 1 (0.5) |
| Increased serum ferritin* | 1 (0.5) |
| Rash | 6 (3.2) |
| Pruritus | 3 (1.6) |
| Alopecia | 1 (0.5) |
| Increased tendency to bruise | 1 (0.5) |
| Prurigo | 1 (0.5) |
| Maculo-papular rash | 1 (0.5) |
| Papular rash | 1 (0.5) |
| Pruritic rash | 1 (0.5) |
| Lenticular opacities | 2 (1.1) |
| Cataract | 1 (0.5) |
| Ocular hypertension | 1 (0.5) |
| Retinal disorder | 1 (0.5) |
| Vision blurred | 1 (0.5) |
| Visual acuity reduced | 1 (0.5) |
| Visual impairment | 1 (0.5) |
| Headache | 5 (2.7) |
| Dizziness | 1 (0.5) |
| Dysgeusia | 1 (0.5) |
| Paraesthesia | 1 (0.5) |
| Deafness | 2 (1.1) |
| Hypoacusis | 1 (0.5) |
| Tinnitus | 1 (0.5) |
| Vertigo | 1 (0.5) |
| Fatigue | 1 (0.5) |
| Granuloma | 1 (0.5) |
| Malaise | 1 (0.5) |
| Chromaturia | 1 (0.5) |
| Glycosuria | 1 (0.5) |
| Micturition urgency | 1 (0.5) |
| Lymphadenopathy | 2 (1.1) |
| Sickle cell crisis | 2 (1.1) |
| Back pain | 1 (0.5) |
| Muscle spasms | 1 (0.5) |
| Bronchial hyperreactivity | 1 (0.5) |
| Pulmonary thrombosis | 1 (0.5) |
| Left ventricular hypertrophy | 1 (0.5) |
| Tuberculosis | 1 (0.5) |
| Lactose intolerance | 1 (0.5) |
| Marrow hyperplasia | 1 (0.5) |
| Abortion spontaneous | 1 (0.5) |
| Hypertension | 1 (0.5) |

*Increased laboratory parameters assessed by investigators to be clinically significant and reported as adverse events. No specific parameters were defined for reporting laboratory assessments as adverse events.

**Table S2. Adverse events leading to deferasirox dose adjustment or interruption, irrespective of study drug relationship**

| **Adverse event, n (%)** | **All patients (*N*=185)** |
| --- | --- |
| Any adverse event | 107 (57.8) |
| Vomiting | 16 (8.6) |
| Nausea | 10 (5.4) |
| Diarrhoea | 9 (4.9) |
| Abdominal pain | 7 (3.8) |
| Upper abdominal pain | 4 (2.2) |
| Abdominal distension | 1 (0.5) |
| Lower abdominal pain | 1 (0.5) |
| Dental caries | 1 (0.5) |
| Flatulence | 1 (0.5) |
| Gastritis | 1 (0.5) |
| Gastrointestinal haemorrhage | 1 (0.5) |
| Lip disorder | 1 (0.5) |
| Oral paraesthesia | 1 (0.5) |
| Retroperitoneal haemorrhage | 1 (0.5) |
| Tooth disorder | 1 (0.5) |
| Toothache | 1 (0.5) |
| Varices oesophageal | 1 (0.5) |
| Increased serum creatinine* | 11 (5.9) |
| Increased serum ferritin* | 10 (5.4) |
| Increased ALT* | 5 (2.7) |
| Increased AST* | 5 (2.7) |
| Increased transaminases* | 5 (2.7) |
| Increased hepatic enzyme* | 2 (1.1) |
| Increased urine protein/creatinine ratio* | 2 (1.1) |
| Abnormal ALT* | 1 (0.5) |
| Abnormal liver function test* | 1 (0.5) |
| Decreased weight* | 1 (0.5) |
| Increased white blood cell count* | 1 (0.5) |
| Pneumonia | 6 (3.2) |
| Upper respiratory tract infection | 5 (2.7) |
| Gastroenteritis | 4 (2.2) |
| Viral gastroenteritis | 4 (2.2) |
| Urinary tract infection | 4 (2.2) |
| Pharyngitis | 3 (1.6) |
| Catheter-related infection | 2 (1.1) |
| Viral infection | 2 (1.1) |
| Appendicitis | 1 (0.5) |
| Catheter bacteraemia | 1 (0.5) |
| Endocarditis | 1 (0.5) |
| Lobar pneumonia | 1 (0.5) |
| Lung infection | 1 (0.5) |
| Mononucleosis syndrome | 1 (0.5) |
| Osteomyelitis | 1 (0.5) |
| Pharyngitis streptococcal | 1 (0.5) |
| Pyelonephritis | 1 (0.5) |
| Acute pyelonephritis | 1 (0.5) |
| Salmonellosis | 1 (0.5) |
| Sinusitis | 1 (0.5) |
| Staphylococcal infection | 1 (0.5) |
| Staphylococcal sepsis | 1 (0.5) |
| Pyrexia | 16 (8.6) |
| Chest pain | 8 (4.3) |
| Catheter site pain | 1 (0.5) |
| Catheter thrombosis | 1 (0.5) |
| Chills | 1 (0.5) |
| Facial pain | 1 (0.5) |
| Influenza-like illness | 1 (0.5) |
| Necrosis | 1 (0.5) |
| Peripheral oedema | 1(0.5) |
| Sickle cell crisis | 23 (12.4) |
| Headache | 11 (5.9) |
| Convulsion | 2 (1.1) |
| Dizziness | 2 (1.1) |
| Altered state of consciousness | 1 (0.5) |
| Encephalopathy | 1 (0.5) |
| Hepatic encephalopathy | 1 (0.5) |
| Hyperaesthesia | 1 (0.5) |
| Intraventricular haemorrhage | 1 (0.5) |
| Somnolence | 1 (0.5) |
| Back pain | 6 (3.2) |
| Pain in extremity | 4 (2.2) |
| Arthralgia | 2 (1.1) |
| Bone cyst | 1 (0.5) |
| Costochondritis | 1 (0.5) |
| Muscle spasms | 1 (0.5) |
| Musculoskeletal pain | 1 (0.5) |
| Osteonecrosis | 1 (0.5) |
| Oropharyngeal pain | 5 (2.7) |
| Dyspnoea | 2 (1.1) |
| Cough | 1 (0.5) |
| Lung infiltration | 1 (0.5) |
| Pleural effusion | 1 (0.5) |
| Pulmonary thrombosis | 1 (0.5) |
| Respiratory failure | 1 (0.5) |
| Sinus congestion | 1 (0.5) |
| Wheezing | 1 (0.5) |
| Cholelithiasis | 5 (2.7) |
| Cholecystitis | 2 (1.1) |
| Chronic cholecystitis | 1 (0.5) |
| Hepatic failure | 1 (0.5) |
| Abnormal hepatic function | 1 (0.5) |
| Rash | 2 (1.1) |
| Pruritus | 1 (0.5) |
| Macular rash | 1 (0.5) |
| Papular rash | 1 (0.5) |
| Vesicular rash | 1 (0.5) |
| Swelling face | 1 (0.5) |
| Urticaria | 1 (0.5) |
| Dysuria | 1 (0.5) |
| Glycosuria | 1 (0.5) |
| Haematuria | 1 (0.5) |
| Oliguria | 1 (0.5) |
| Proteinuria | 1 (0.5) |
| Renal colic | 1 (0.5) |
| Acute renal failure | 1 (0.5) |
| Renal impairment | 1 (0.5) |
| Acute chest syndrome | 2 (1.1) |
| Anaemia | 1 (0.5) |
| Hypersplenism | 1 (0.5) |
| Spleen disorder | 1 (0.5) |
| Dehydration | 1 (0.5) |
| Hypokalaemia | 1 (0.5) |
| Hypovitaminosis | 1 (0.5) |
| Iron overload | 1 (0.5) |
| Hypertension | 2 (1.1) |
| Haematoma | 1 (0.5) |
| Jugular vein distension | 1 (0.5) |
| Vision blurred | 2 (1.1) |
| Retinal disorder | 1 (0.5) |
| Visual acuity reduced | 1 (0.5) |
| Alcohol poisoning | 1 (0.5) |
| Drug toxicity | 1 (0.5) |
| Incision site pain | 1 (0.5) |
| Pregnancy | 3 (1.6) |
| Cholecystectomy | 2 (1.1) |
| Hip arthroplasty | 1 (0.5) |
| Congestive cardiac failure | 1 (0.5) |
| Left ventricular hypertrophy | 1 (0.5) |
| Tinnitus | 1 (0.5) |
| Vertigo | 1 (0.5) |
| Breast swelling | 1 (0.5) |
| Priapism | 1 (0.5) |
| Marrow hyperplasia | 1 (0.5) |
| Abnormal behaviour | 1 (0.5) |

*Increased laboratory parameters assessed by investigators to be clinically significant and reported as adverse events. No specific parameters were defined for reporting laboratory assessments as adverse events.
